# Comparison of *Schmallenberg* virus sequences isolated from mammal host and arthropod vector

**DOI:** 10.1007/s11262-018-1607-7

**Published:** 2018-10-19

**Authors:** Julia Kęsik-Maliszewska, Aleksandra Antos, Jerzy Rola, Magdalena Larska

**Affiliations:** Department of Virology, National Veterinary Research Institute, Al. Partyzantów 57, 24-100 Puławy, Poland

**Keywords:** *Schmallenberg* virus (SBV), Phylogenetic study, Poland, *Culicoides*, Vector

## Abstract

*Schmallenberg* virus (SBV) is the member of Peribunyaviridae family, which comprises pathogens of importance for human and veterinary medicine. The virus is transmitted only between animals and mainly by biting midges of the genus *Culicoides*. This study was performed in order to determine SBV genetic diversity and elucidate the host–vector adaptation. All three viral segments were analysed for sequence variability and phylogenetic relations. The Polish SBV strains obtained from acute infections of cattle, congenital cases in sheep, and from *Culicoides* midges were sequenced using Sanger and next-generation sequencing (NGS) methods. The obtained sequences were genetically similar (99.2–100% identity) to the first-detected strain BH80/11—4 from German cattle. The sampling year and origin of Polish sequences had no effect on molecular diversity of SBV. Considering all analysed Polish as well as European sequences, ovine-derived sequences were the most variable, while the midge ones were more conserved and encompassed unique substitutions located mainly in nonstructural protein S. SBV sequences isolated from *Culicoides* are the first submitted to GenBank and reported.

**Electronic supplementary material:**

The online version of this article (10.1007/s11262-018-1607-7) contains supplementary material, which is available to authorized users.

## Introduction

*Schmallenberg* virus emerged as an unknown *orthobunyavirus*, causing first unspecific signs of fever and diarrhoea in adult cattle in Germany and the Netherlands in 2011, which later unraveled into more serious outbreaks of foetal malformations, stillbirths and abortions. The viruses of *Orthobunyavirus* genus, Peribunyaviridae family, are transmitted by vectors such as mosquitos, biting midges and ticks. Some, such as *Oropouche* virus (OROV), cause human febrile illness in South America, while other, such as *Akabane* virus (AKAV), Aino virus (AINV) and Peaton virus (PEAV), infect animals causing abortions, stillbirths and malformations in domestic ruminants in Australia, Asia and Africa. SBV-specific antibodies were detected in most domestic, wild and exotic ruminants, camelids, wild boars, and even in dogs. The virus is transmitted by *Culicoides* Latreille (Diptera: Ceratopogonidae), which are also known vectors of other emerging viruses such as Bluetongue (BTV) or African horse sickness (AHSV). Due to the lack of mandatory notifications of SBV-suspected cases, the general impact of SBV on European animal trade remains uncertain; however, during the outbreaks seroprevalence reached up to 100%. At the moment, Poland as most European countries is going through a secondary wave of SBV epidemics [1–3].

SBV genome consists of the three negative single-stranded RNA segments. The large (L) segment encodes RNA-dependent RNA polymerase (RdRp); medium (M) segment encodes a polyprotein precursor, which is post-translationally cleaved into nonstructural protein (NSm) and two glycoproteins (Gn and Gc); and small (S) encodes nucleocapsid (N) protein and a nonstructural protein (NSs) in an overlapping reading frame. Gc and Gn proteins are present on the surface of the virion inserted in the lipid envelope, which makes them the target of host antibodies [4–6]. They play a role in viral attachment and cell fusion [7]. In a few studies, M segment was described as the most divergent part of SBV genome with the hypervariable region (HVR) encoding N-terminus of Gc protein [8–10]. The N protein is responsible for creating viral envelope, and is considered as the best antigen for serological and molecular SBV testing. The NSs is a major virulence factor for orthobunyaviruses [10], but is nonessential to virus viability in mammalian and insect cells [11].

Hitherto, the phylogenetic analyses within SBV were rather limited to S segment studies and a small amount of data originating only from the ruminant host [12–15]. Previous papers presented no geographic- or limited time-dependent variability of SBV genome [1]. In order to extend the knowledge on the diversity of the novel virus as well as to elucidate the host-vector adaptation of SBV, comparative genetic analysis of new Polish plus beforehand reported European SBV sequences was performed in relation to different genome fragments and different host species including, for the first time, *Culicoides* vector.

## Materials and methods

### Sample origin and RNA purification

The SBV-positive samples initially included in the study were collected between 2012 and 2013 from central nervous system tissue of 27 aborted ovine foetuses; 20 serum samples from cattle herds during acute infection from Silesia and West Pomeranian provinces [16, 17]; a serum sample from elk from Podlasie province [18]; and three positive samples of bull semen collected at Warmia-Masuria province [19]. The *Culicoides* were collected during BTV virus monitoring program using UV light traps placed at the cattle farms throughout the country between 2012 and 2015. Collected insects were entomologically examined, segregated and pooled (approx. 23 individual females) according to their species and parity status (depending on feeding and oviposition). Since *C. obsoletus* and *C. scoticus* females cannot be distinguished morphologically, those pools were marked as *C. obsoletus*/*scoticus* complex. Sixty-seven SBV-positive pools from the most abundant *Culicoides obsoletus*/*scoticus* complex or *Culicoides punctatus* species were found. More pools were parous females (with pigmented abdomen, which blood fed and oviposited at least once), than gravid (containing a mature egg batch in abdomen) and freshly blood fed. See Supplementary Table 1 for detailed list of samples successfully sequenced.

Ten percent mammal tissue homogenates were prepared in RLT lysis buffer (Qiagen, Germany) using Lysing Matrix D Tubes (MP Biomedicals, France) and FastPrep-24 instrument (MP Biomedicals, USA) for 90 s at 6.5 m/s speed. The insects were drained from 70% ethanol in which they were previously stored, and homogenized in 500 µl RLT using the same system. Subsequently, total RNA was extracted using RNeasy Mini Kit (Qiagen, Germany) in the QIAcube automatic purification station (Qiagen, Germany). Total RNA from serum was extracted using TRI Reagent (Sigma-Aldrich, USA) and eluted in a final volume of 20 µl of nuclease-free water. Semen was extracted using Viral RNA Mini Kit (Qiagen, Germany) after Qiazol Lysis Reagent (Qiagen, Germany).

### Conventional reverse transcription PCR (RT-PCR), molecular cloning and sequencing

RT-PCR was conducted using Transcriptor One-Step RT-PCR Kit (Roche, Switzerland). Complementary DNA (cDNA) of three SBV segments was amplified using 13 primer pairs kindly provided by Dr. Wernike from Friedrich Loeffler Institut (FLI) Insel Riems, Germany [9] and one pair of self-designed primers (forward: TGACATTCCATGAGTCTATG and reverse: GTCGGATTGTCTCCTGCAAAC flanking from 668 to 1874 nt in segment L). Amplification products were analysed in agarose gels, gel purified using QIAquick Gel Extraction Kit (Qiagen, Germany) and subjected to DNA Sanger sequencing by external company using BigDye Terminator kit v3.1 Kit and Cycle Sequencing 3730XL Genetic Analyzer (Applied Biosystems, USA). The cDNA amplicons were purified by the QIAquick PCR Purification kit (Qiagen, Germany), followed by sequencing in the ABI PRISM 3100 Genetic Analyzer (Applied Biosystems, USA), and the results were analysed by GeneScan Analysis Software (Applied Biosystems, USA). For samples that contained insufficient amount of RNA to obtain nucleotide sequences, molecular cloning of gel purified DNA fragments prior to sequencing was performed using Qiagen PCR Cloning Plus Kit with pDrive cloning vector (Qiagen, Germany) and *Escherichia coli* cells (Qiagen, Germany). Purification of plasmid DNA was performed with Pure Link Plasmid Miniprep Kit (Thermo Fisher Scientific, USA). The presence of insert was confirmed by PCR using Jump Start AccuTaq LA DNA polymerase (Sigma-Aldrich, USA).

### Next-generation sequencing (NGS)

NGS for two samples was performed. Isolate PL_13_130-6_ovis was isolated from brain tissue of stillbirth lamb. Virus was propagated in BHK-21 (baby hamster kidney) cell culture. The viral RNA was extracted after the third passage, while the RNA of the second SBV strain PL_12_1018-08_bov was extracted directly from the serum of infected cow. Libraries for NGS were prepared using NEBNext Ultra RNA Library Prep Kit for Illumina and then checked in BioAnalyzer 2100 using Agilent High Sensitivity DNA Kit. Illumina sequencing was performed with MiSeq Reagent Kit v2 mode PE250. Bioinformatic analysis was carried out using trimmomatic (filtering reads, trimming adapters) and CLC GenomicWorkbench (mapping reads to reference sequences and De novo assembly of NGS data).

### Phylogenetic analysis

Alignments of the nucleotide consensus sequences and deduced amino acid (aa) sequences and comparative sequence analysis were carried out with MEGA version 5.2, BioEdit v. 7.2.5 and Clustal Omega. Primers were designed using Primer3 v.0.4.0 and OligoAnalyzer 3.1. Obtained sequences of Polish isolates were submitted to GenBank (Supplementary Table 1). The ratio between nonsynonymous and synonymous substitutions (dN/dS) was used to estimate the patterns of selection [20]. The higher the ratio, the higher positive selection onto the protein. Conversely, the ratio below 1 suggests the protein evolves slowly under negative (purifying) selection, meaning more conservative. Neutral evolution occurs when dN/dS ~ 1.

## Results

### Phylogenetic analysis of Polish SBV sequences

Seven fragments of SBV detected in lamb brain tissue samples (C_t_ cut off 19.2–31.3) were obtained: three segment S, two segment M and one segment L as well as one fragment containing HVR corresponding to 410–687 aa of M polyprotein (Supplementary Table 1). From cattle serum (C_t_ cut off 22.5–27.9), six sequences were assembled: one full S and M segment, additionally two HVR sequences and two complete segment L. Unfortunately, the attempts to sequence the virus from elk serum and bull semen failed.

**Fig. 1:**
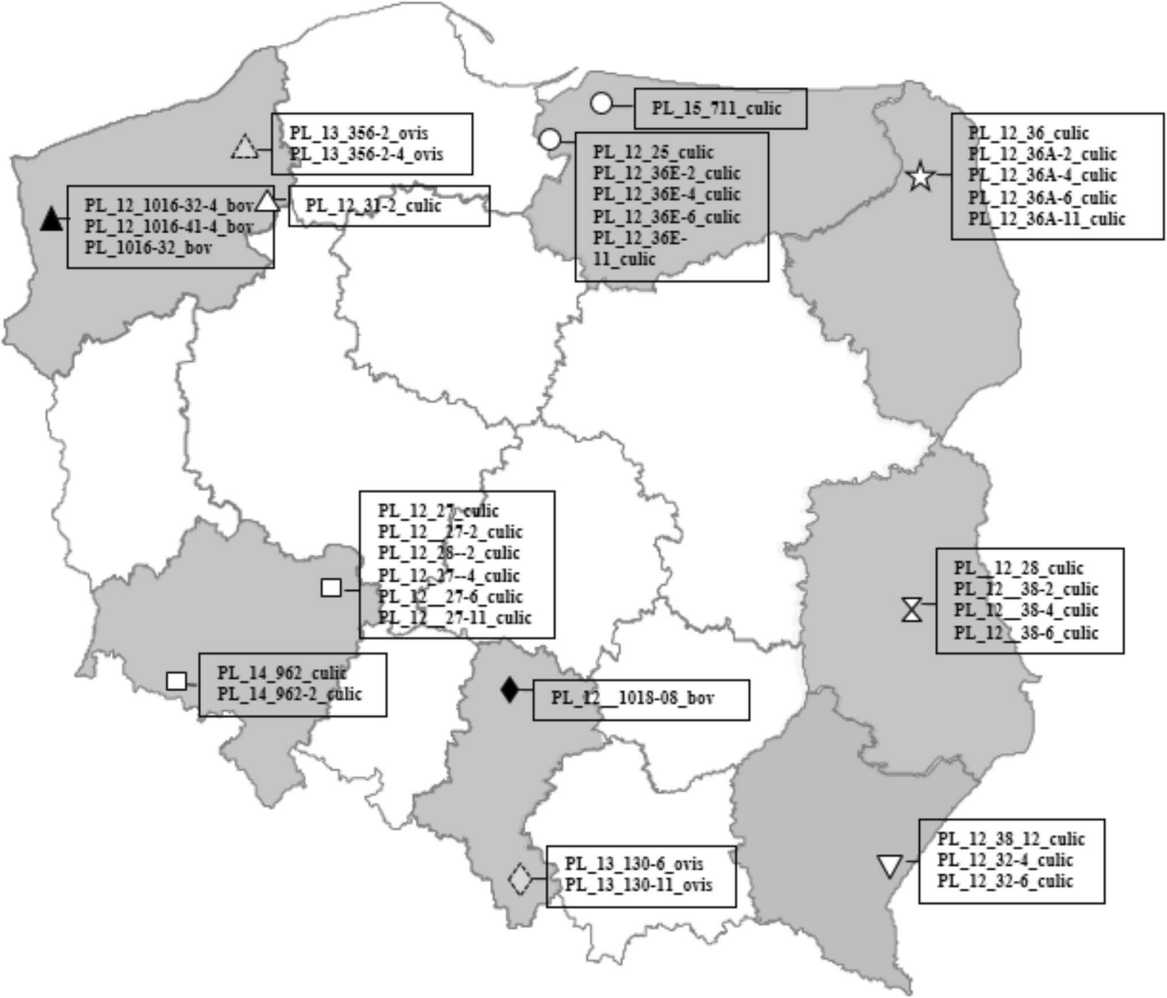
Map of geographical location of Polish isolates from the present study. To follow the association between localization and molecular diversity of isolates, they were marked with the same symbols on the phylogenetic trees

Twelve of 44 SBV-positive pools from 2012 (C_t_ 17.6–29.0), four of 13 from 2014 (C_t_ 22–39.3) and one (C_t_ 29.3) of 10 positive in 2015 provided seven segment S sequences, one full segment M sequence as well as five sequences within HVR, 7 Gn (1–188 aa) and five Gc (1141–1394 aa) encoding fragments. Only three short SBV sequences of L segment (1137–1390 aa) were obtained from the midge. The geographical distribution of the animals which provided the sequences is presented in Fig. 1.

**Fig. 2:**
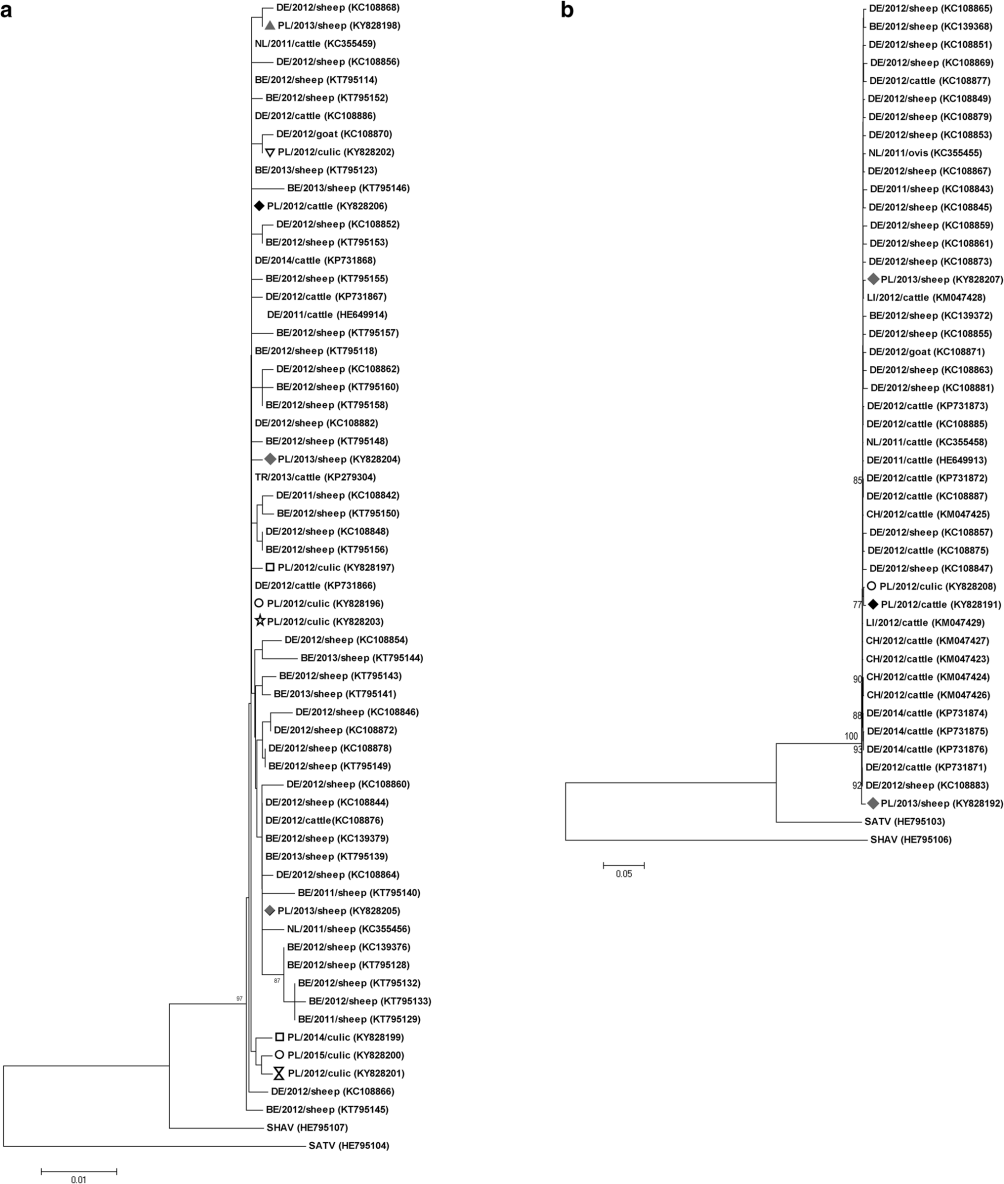

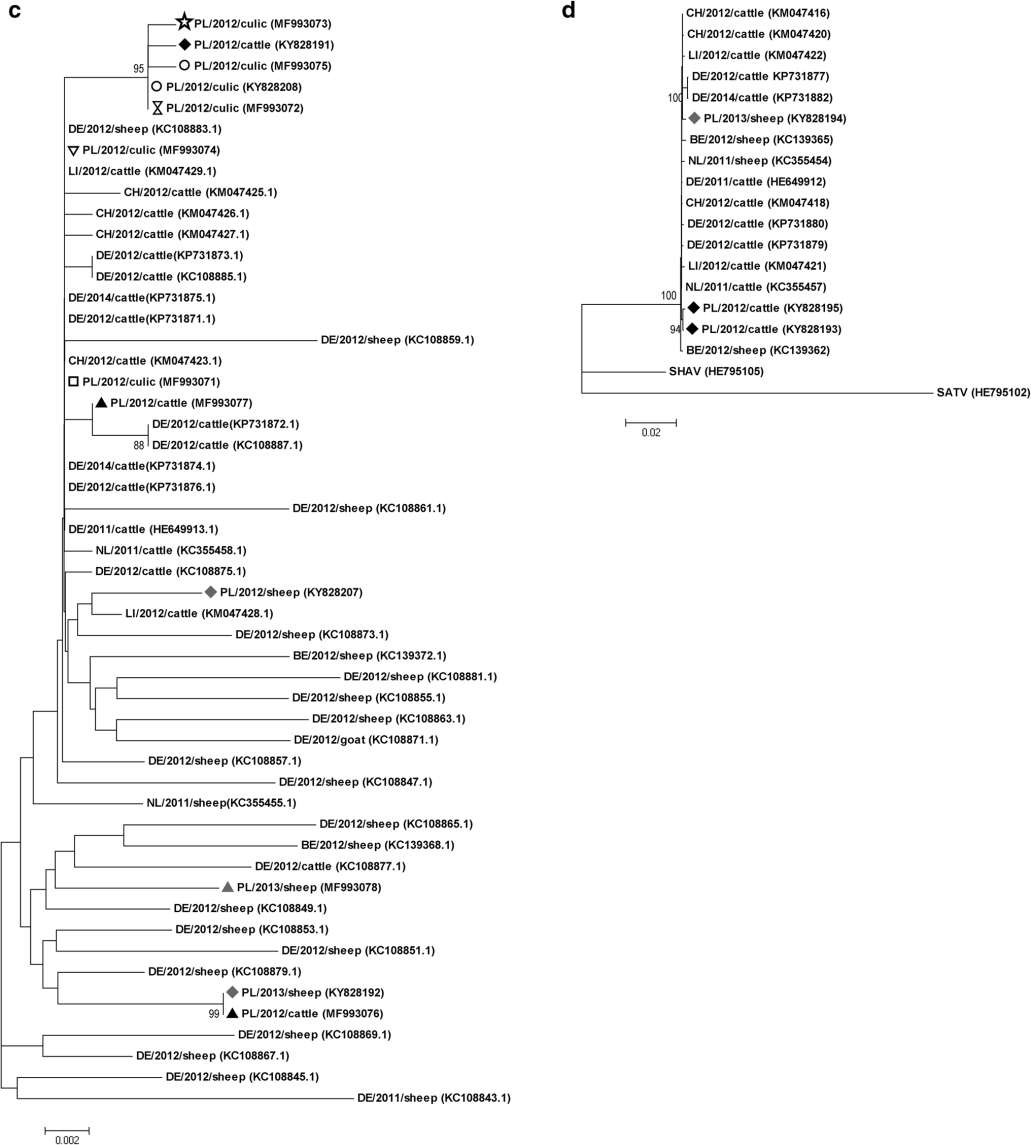
Phylogenetic trees of complete segment S (**a**), segment M (**b**), hypervariable region (HVR) corresponding to 410–687 aa protein in M segment (**c**), and complete segment L (**d**) nucleotide sequences constructed using maximum likelihood method, Kimura 2-parameter model with 1000 bootstrap replication. Polish strains marked with different shapes corresponding to geographic origin (Fig. 1). SBV reference sequences representing different countries of origin from different animal species were obtained from GenBank (Supplementary Table 2) and indicated by accession numbers. Numbers at nodes represent percentage of 1000 bootstrap replicates (values < 70 were not shown). Scale bars indicate nt substitution per site. All the strains are marked with their ISO country code, year of detection and animal species. GenBank accession numbers in parenthesis

Among Polish sequences, only 0–2 nucleotide (nt) changes per sequence were observed within segment S sequence, which would result in 0–1 amino acid (aa) substitutions in N protein and 0–2 amino acid substitutions in NSs protein. The S segment nucleotide sequences were highly homologous among Polish isolates (99.3–100% identity) and to German reference strain BH80/11—4 (99.6%-100%), first SBV strain ever identified [19]. Phylogenetic tree of S segments reflected virus genetic stability (Fig. 2a). Most of Polish sequences were distributed throughout the tree, regardless of the origin or year of detection, showing seemingly random relationship to Belgian and German sequences. However, three strains originating from *Culicoides obsoletus*/*scoticus* complex midges (PL_12_28_culic, PL_14_962_culic, PL_15_711_culic) formed separate branch. In general, sequences derived from particular host, country or sampling year did not form any clear clades or subgroups either.

In M segment, sequence similarity between Polish isolates and reference strain BH 80/11—4 presented 99.2–99.7% identity and among Polish isolates was 98.6–99.9%. Comparison of 12 Polish and reference HVR sequences showed 57 nt substitutions; 35 of them were nonsynonymous (dN/dS ratio 1.6). Due to the low diversity of segment M, phylogenetic trees constructed on the complete segment or HVR fragment sequences showed again low resolution (Fig. 2b, c). On HVR fragment and full-length M segment trees comprising the same available sequences, four out of six sequences derived from *Culicoides* clustered together with sequence from the first SBV outbreak in cattle herd in Poland creating separate branch from the rest of the sequences. Other Polish sequences were spread across the tree showing highest affinity to German, Swiss and Liechtenstein sequences. The same as for S segment, the origin, year of detection of the Polish strains and animal species had no effect on the pattern of molecular diversity of SBV in our country.

The analysis of aligned sequences has shown that segment L is genetically stable (99.9–100% identity with reference strain BH80/11—4). Among Polish sequences, 24 nt substitutions (eight per sequence) of which four (1.3 per sequence) were nonsynonymous (Asp121Asn, Met1093Thr, Glu1159Gln, Glu1823Lys) were found. On the phylogenetic tree based on L segment, two bovine isolates from Silesia province formed separate branch from the rest of the sequences.

### Comparative analysis of SBV sequences isolated from mammal host and arthropod vector

The limited number and spatio-temporal divergence of Polish sequences obtained in the presented study was insufficient to investigate SBV variability in different hosts. Representative reference nucleotide sequences from different countries, studies and species available in GenBank were selected to supplement the analysis (Supplementary Table 2). Only sequences encoding complete ORF were chosen for detailed analysis. Sequences that were identical, originating from the same study, country and species were excluded. If sequences were derived from tissues as well as passaged in cell lines—only ones from original material were chosen. Finally, 62 sequences (11 Polish, 51 European) of segment S, 45 (4 Polish, 41 European) of M segment and 17 (3 Polish, 14 European) coding region of L segment were included in the analysis. To study N-terminal sequence of Gc known as the most variable fragment in whole SBV genome (HVR), 52 fragments (12 Polish, 40 from GenBank) were selected. Previous studies had defined the HVR located in Gc protein between 457 and 847 aa [8] or between 493 and 629 aa [9]. In this study, the HVR was slightly modified into a fragment between 410 and 687 aa to present substitutions in the, so far unique, sequences obtained from *Culicoides*. The genome of the first SBV strain BH80/11—4 detected in German cattle was used as a reference in all the analyses [21].

**Fig. 3:**
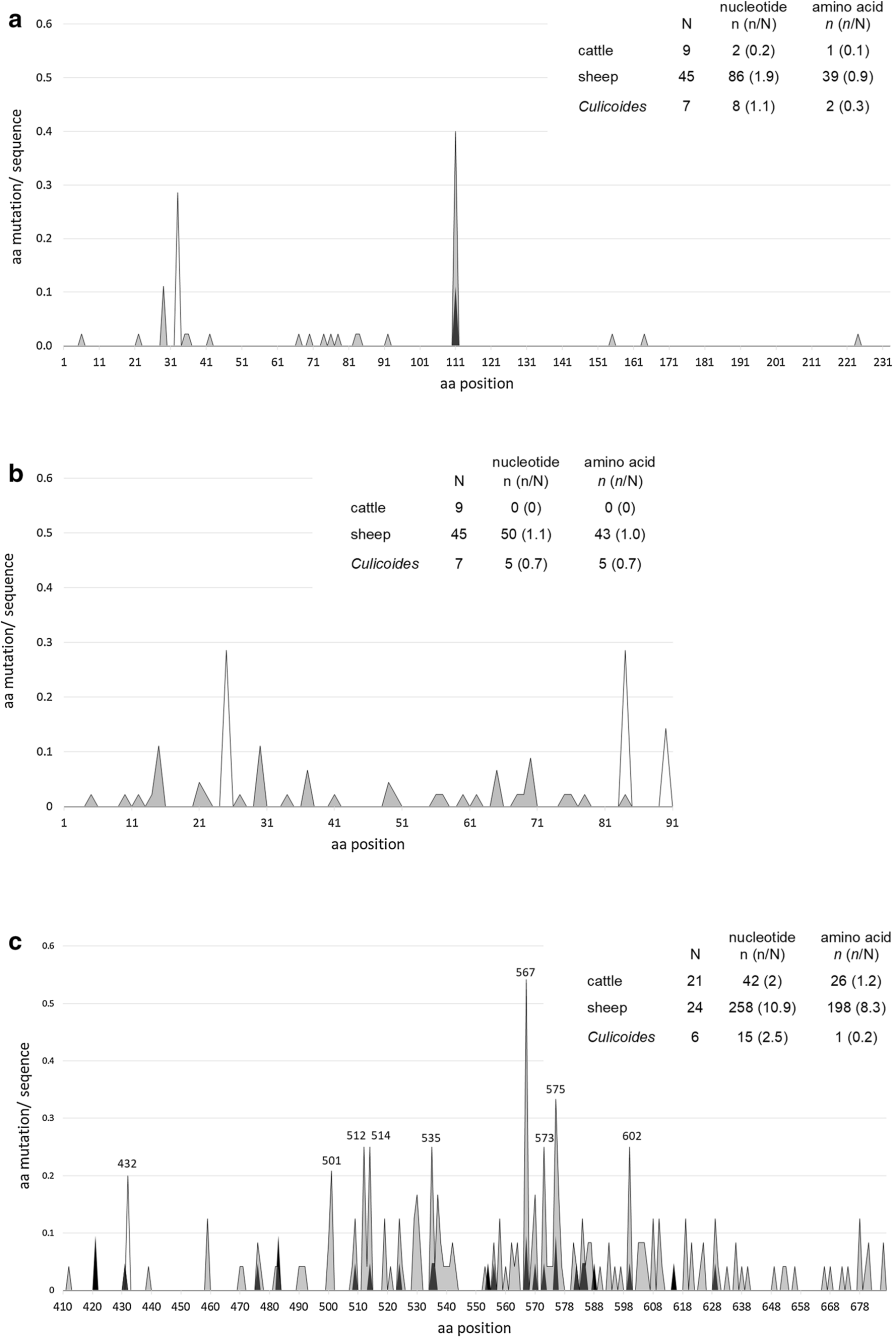
Distribution of aa substitutions or indel mutations within deduced aa sequences of the most variable of viral proteins: nucleoprotein (**a**), nonstructural protein S–NSs (**b**), and HVR of glycoprotein C–Gc (**c**) with respect to different hosts. In the tables, number of nucleotide (n) or amino acid (*n*) variation was divided per number (N) of sequences from respective species included in the analysis (including Polish and reference sequences). The goat sequence was excluded. The black arrow represents two aa insertions

Comparing nucleotide changes in all European and Polish sequences obtained from different species, we can conclude that N protein is the most conserved among cattle (Fig. 3a). In *Culicoides*-derived sequences, however limited in number, less substitutions per sequence were observed. In sheep, sequence substitutions were the most frequent. Interestingly, nucleotide substitutions observed in SBV isolates from *Culicoides* did not occur in mammalian strains. G354A substitution (Ser111Asn) noted in one bovine sequence was observed also in approximately 38% sheep isolates, which may determine this amino acid the most variable among N protein. NSs protein among cattle strains proved to be the most conserved region, where no nt or aa changes occurred (Fig. 3b). In arthropod sequences, all nt changes were nonsynonymous and were unique to the sequences originating from mammals: Asn25Asp, His84Arg, Asp90Gly. However, His in 84 position was also substituted to Tyr in sheep-derived sequence from Belgium (GenBank Accession Number—KT7951461). The most mutations occurred in sheep-derived sequences, including seven substitutions leading to truncated NSs sequence. Variability in all analysed sequences of N protein was 99.9–100%, while of NSs 98.9–100%. The analysis of the nonsynonymous-to-synonymous substitution ratio (dN/dS) revealed that nucleoprotein is much more conserved than NSm S, respectively, 0.8 (43 nonsynonymous/55 synonymous) and 6.1 (49/8).

Our analysis confirmed that, as described for other orthobunyaviruses, M segment was the most variable segment in SBV genome. Comparing all analysed sequences, identity of Gn was 99.2–99.9%, of NSm − 99.4 to 100% and of Gc − 99.0 to 99.9%. Nucleotide sequence analysis revealed that, as well as in S segment, sequences obtained from sheep were more variable than those from cattle (respectively 22.3 and 8.5 nt per sequence, resulting in 16.5 and 4 aa substitution per deduced aa sequence). The overall dN/dS ratio reached 2.1 (479/233) for all analysed sequences. However, it was significantly higher for ovine sequences − 2.9 (380/133) than for bovine ones − 0.9 (79/90), which suggested positive selection only in sheep-derived sequences. One complete M segment sequence from *Culicoides* with only four and one from goat sequence with 16 aa substitution did not allow to draw firm conclusions.

Comparative analysis of all deduced amino acid sequences (*n* = 52) showed that positive selection pressure on HVR in Gc protein is more marked in sheep (dN/dS 3.3) than in cattle (dN/dS 1.6), while in *Culicoides* only negative selection occurred (dN/dS 0.1) (see Fig. 3c). This reflected sequence similarity for sheep, cattle and *Culicoides* sequences to the reference sequence, respectively, 97.9–100%, 98.7–100% and 99.9–100%. In one available goat sequence, 9 nt substitution arose, all nonsynonymous (99% identity with reference strain) from which only two were detected also in sheep from the same study (9). Nonsynonymous-to-synonymous substitution ratio for all sequences in HVR was 2.6 and differed slightly from calculated for the complete segment M (2.1). Accumulation of nonsynonymous mutations in sequences from mammalian host occurred between 470 and 640 aa (Fig. 3c). A single aa change (Arg432Ile) was observed in only one PL_12_36_culic strain originating from *C. obsoletus*/*scoticus* complex pool and was unique among other sequences. The most frequent aa variation was Gln567Lys which was observed in half ovine and one-tenth of bovine SBV sequences. Second, most common change was observed in 575 aa, where Phe was replaced by Ser in 10% of sequences from cattle and 29% from sheep and by Pro in one sheep sequence. The indel mutations were only observed in HVR, which included insertion of Ile and Phe (between 574 and 575 aa in sheep sequence, GenBank accession number—KC1088591) and the deletion of 12 aa (from 532 to 543 in sheep sequence KC1088531) [9].

The detailed analysis of M segment in arthropod vector included eight sequences of 12–564 nt (1–188 aa) fragment which corresponded to N-terminal fragment of Gn and six sequences of 3465–4195 nt fragment overlapping Gc (1141–1394 aa). In both fragments, sequences were highly conserved with 100% and 99.6–100% homology, respectively. The substitutions observed in Gc fragment isolated from Culicoides were not described in any mammal sequence: nonsynonymous change G3757C (S1249T) in one strain and synonymous A3920G in two strains.

In L segment, comparison of all analysed 17 sequences revealed 79 nt (6.1 per sequence) and 13 aa (1 per sequence) substitutions in sequences from cattle, as well as 34 nt (8.5 per sequence) and 13 aa (3.25 per sequence) in sequences from sheep. The dN/dS ratio was only 0.2 and 0.6 for bovine and ovine sequences, respectively, suggesting negative selection in this segment. In all sheep sequences, a difference (Glu/Gln or Glu/Asp) at 1159 position in respect to reference strain was observed. Unfortunately, only three fragments (1137–1390 aa) from segment L were obtained from insect-derived material. However, the analysed fragment seemed to be highly homological among the isolates (99.9–100% identity to reference strain). Two point mutations (T3721C, T4012C) occurred resulting in one aa substitution (Tyr1236His) in one culicoid sequence (PL_12_27—11_culic) from *C. obsoletus*/*scoticus* pool, while no changes were observed in the remaining two.

## Discussion

Collective analysis of Polish, as well as available European SBV isolate sequences, confirmed high genetic stability of S and L segments in the mammal host and presumably in arthropod vector. As described previously, segment M containing glycoprotein C hypervariable ectodomain was the most variable fragment of viral genome [9, 10]. Still, comparing to better known *orthobunyavirus* of veterinary importance—*Akabane* virus, SBV presented 25 times higher mutation rate, as it is understood for the virus in its epidemic stage [22]. Our results have revealed some quantitative and qualitative differences of nt as well as aa mutations mostly in ruminants. Interestingly, the sequences derived from *Culicoides*, which should be emphasized that these were described for the first time, seemed less variable. Characteristically, the ovine SBV sequences in all viral segments were the most divergent, including sequences originating from the same sheep flock, which differed significantly. Furthermore, some researchers reported cases of two or three SBV variants found in single animal, which with respect to overall low variability of SBV resulted more probably from co-infection with different SBV strains [12, 23]. Such situations were not reported in cattle. Interestingly, SBV in sheep is more pathogenic causing important loses in breeding but the seroprevalence is lower than in cattle [24]. It may be speculated that in sheep SBV multiplies more efficiently or it interacts differently with ovine immune defense, and thus antigenic escape, expressed in mutations, results in higher morbidity than in cattle. SBV is transmitted to vertebrae host by an insect vector and direct transition between mammals is unlikely. Therefore, the differences in SBV sequences obtained from insect and vertebrae host may refer to formation of virus quasispecies as reported for other virus vector systems [25]. To unravel this, the comparison of nucleotide sequences originating from host and vector from one disease outbreak would be necessary.

Based on evolutionary and phenotypic studies on arboviruses, it was concluded that they evolved from arthropod-specific ancestors [26]. Assumed high genetic stability in arthropod vector may be the result of SBV better and more conserved adaptation to counteracting insect host immune defense and virus replication in insects. The argument for this adaptation may be the fact that peribunyaviruses that are teratogenic in vertebrae are harmful to developing arthropod oocyte and embryo at the same time [27]. On the other hand, it seems that the virus does not replicate in the insects as efficiently as in mammal host, meaning lower number of transcriptions equals fewer RNA polymerase mistakes and thereof nucleotide mutations. As discussed earlier [28], bimodal distribution of C_t_ values in *Culicoides* pools may result from different patterns of virus replication: transmissible, active virus multiplication in parous and gravid females (C_t_ around 22) and sub-transmissible, probably persistent (C_t_ around 35) infection in nulliparous or passive carriage of the virus in *C. punctatus*. This finding may be supported by experiments on *C. sonorensis* with BTV where insects with infectious virus had lower C_t_ values than those with persistent, sub-transmissible infections or passively carrying the viral RNA in engorged blood [29]. It was also reported that viral transcription rate of La Crosse virus (LACV) is strictly connected and co-regulated with mosquito cell transcription due to cap snatching occurring in all bunyaviruses. Insects in diapause dramatically reduce metabolic activity and cell replication. It could modulate virus replication favouring persistent infections and protect vector from virus detrimental effect during dormancy and thus enhancing the overwintering efficiency of LACV [27]. In our study, NSs was the most variable SBV protein in *Culicoides* SBV strains. All detected substitutions were nonsynonymous and independent from the mammalian ones. This could suggest specific role of this protein in infection of arthropod vector and reflect virus adaptation to *Culicoides* immune defense. NSs protein counteracts the immune system of vertebrae host. It shuts down antiviral protein, such as IFN alpha, and regulates translation, apoptosis and RNA polymerase II [11]. In arthropod vector, NSs is also suspected to shut down insect immune response; however, details are still unknown [30]. In *Bunyamwera* virus (BUNV), NSs sequence is required for virus replication in vitro and in vivo [31]. However, some naturally occurring insect-borne orthobunyaviruses with truncated or lacking NSs were reported [32]. This protein may be crucial for virulence in insects, and hence in plant-infecting *Tospovirus* (*Bunyavirales* order) called Tomato spotted wilt virus, thrips infected with NSs-lack mutant viruses were not able to transmit and amplify the virus efficiently [33]. In LACV and BUNV, NSs does not affect RNA polymerase II or inhibit protein synthesis, as it does in mammalian cells, thus establishing persistent infection instead of apoptosis [27, 31, 34]. Apoptosis of the arbovirus-infected insect cells can have strong antiviral effect, but may also reduce vector vitality necessary for efficient feeding on mammal host and transmitting the virus. Therefore, in competent vector apoptosis is suspected to be inhibited or in low level favouring persistent infection [35]. Moreover, researches presented different effect of NSs on replication in cell lines isolated from distinct insect species [31]. It may be speculated that variation in NSs of *Culicoides*-derived SBV strains may be the result of virus adaptation to new European species of insect vectors.

As discussed by Coupeau et al. [12], NSs protein may not be essential in congenital infections in ruminants since NSs-truncated sequences occurred in lambs. Such shortened NSs was detected in lambs from Germany in 2011 and 2012 [9], Belgium in 2012 [12] and in Great Britain in 2012 and 2017 [1]; in Polish sequences such deletions were not observed. Experiments with SBV NSs deletion mutants indicated no virus replication or clinical disease in cattle [11]. NSs is efficient in shutting down most known major antiviral proteins, including INF [30, 36]. Research on ovine foetuses infected with Cache Valley Virus revealed upregulation of some INF type I-induced proteins involved in bunyavirus immune defense [37]. It proves that foetuses in vulnerable gestation period produce an immune response against bunyavirus infection controlled by NSs protein, and hence efficient replication of NSs-truncated mutants in congenital cases needs elucidation. Probably the answer may be hyperinfection reported in some of these cases [12].

Segment M coding Gc, NSm and Gn is proposed as the most variable within whole SBV genome. The N-terminus of Gc protein encodes ectodomain exposed in the virion envelope and is not essential for virus replication in tissue culture, while the C-terminus contains fusion peptide [7]. The identified HVR is probably a result of viral evasion of host immunity [9] and Gc adaptation to new host to get a better entry into host cells [10]. The M segment (precisely Gc) could also be a virulent factor and is involved in host protein shutoff as described by Varela et al. [10]. Greater variation in HVR in sheep may result from the enhanced teratogenic effect observed in this species. Postulated N-linked glycosylation sites in Gc (493, 686, 1353 aa) remains stable in most analysed sequences [23]. However, mutation in 686 aa (from Asn to Asp) in HL-1 atypical isolate [23] was also found in BH200/12—2 [9] both from lamb’s brain.

The HVR of Gc protein in arthropod-derived sequences presented almost no mutations in contrast to mammalian-derived sequences. Possibly lack of immune system pressure and arms race on the Gc ectodomain proves this protein does not play essential role in vector evasion, virulence or adaptation to arthropod host. Probably, as defined for LACV, Gn glycoprotein instead of Gc plays a role in attachment to mosquito cells [38]. Gn glycoprotein is responsible for folding, heterodimerization, and intracellular trafficking of both Gn and Gc proteins [39]. The analysis of N-terminus of Gn, where functional signal peptide for membrane translocation is localized [40], revealed high stability of this glycoprotein fragment in the samples from *Culicoides*.

NSm M takes part in protein synthesis shutoff [41], virus assembly and budding. Mutant lacking NSm can replicate in cell culture but has reduced virulence in mice, in cattle produce viremia and seroconversion [11]. In Rift Valley fever virus (RVFV), deletion of NSm reduced infection, dissemination (possibly by modulation of a mosquito midgut infection barrier) and transmission rates in mosquito proving the essential role of NSm in viral-vector interactions [2, 42]. In Polish Culicoides-derived sequences, similarity was reaching 99.8% with only one synonymous mutation.

RdRp located in the L segment has four conserved regions among peribunyaviruses [43]. Regions 1 and 2 located on amino termini and region 3 with polymerase module (pre-motif A, motif A to E) and region 4 downstream. As in other studies, SBV L segment revealed high stability due to its crucial function in virus replication. As in residual SBV segments, sheep-derived sequences had more mutations than those isolated from cattle.

Analysis of Polish together with European SBV sequences confirmed that the most variable region of the virus genome is N-terminus fragment in Gc within M segment. High sequence identity among strains may simplify the diagnostic testing and the implementation of vaccination scheme. The virus genetic stability found in vector-derived sequences may be the result of prominent virus adaptation and speculated low replication potential in insect organism, while high mutation rate in sheep may coexplain high morbidity in this species. To our knowledge, this is the first report of SBV sequences isolated from the insect vector.

## Electronic supplementary material

Below is the link to the electronic supplementary material.

Supplementary material 1—Table 1 List of characterized Polish sequences from the present study (DOC 57 KB)

Supplementary material 2—Table 2 List of reference sequences used in the study (DOC 88 KB)
