# Supplementary material for: Comparison of Schmallenberg virus sequences isolated from mammal host and arthropod vector
Source: Virus Genes. 2018 Oct 19;54(6):792–803. doi: 10.1007/s11262-018-1607-7 (PMC6244546; doi:10.1007/s11262-018-1607-7)
Supplement: Supplementary file 1 — Supplementary material 1—Table 1 List of characterized Polish sequences from the present study (DOC 57 KB) [file 11262_2018_1607_MOESM1_ESM.doc]

| Segment | Isolate | Material; sampling year | Sequencing method | GenBank accession No. |
| --- | --- | --- | --- | --- |
| S (complete) | PL_12_1018-08_bov | cattle serum; 2012 | NGS | KY828206 |
|  | PL_13_130-6_ovis | lamb brain II passage BHK-21; 2013 | NGS | KY828205 |
|  | PL_13_130-11_ovis | lamb brain; 2013 | Sanger | KY828204 |
|  | PL_12_25_culic | *C. obsoletus/scoticus* complex parous; 2012 | Sanger | KY828196 |
|  | PL_12_27_culic | *C. obsoletus/scoticus* complex parous ; 2012 | Sanger | KY828197 |
|  | PL_12_28_culic | *C. obsoletus/scoticus* complex parous; 2012 | Sanger | KY828201 |
|  | PL_12_38_12_culic | *C. obsoletus/scoticus* complex parous; 2012 | Sanger | KY828202 |
|  | PL_12_36_culic | *C. punctatus* gravid; 2012 | Sanger | KY828203 |
|  | PL_13_356-2_ovis | lamb brain; 2013 | Sanger | KY828198 |
|  | PL_14_962_culic | *C. obsoletus/scoticus* complex blood fed; 2014 | Sanger | KY828199 |
|  | PL_15_711_culic | *C. obsoletus/scoticus* complex parous; 2015 | Sanger | KY828200 |
| M (complete) | PL_12_1018-08_bov | cattle serum; 2012 | NGS | KY828191 |
|  | PL_13_130-6_ovis | lamb brain II passage BHK-21; 2013 | NGS | KY828192 |
|  | PL_13_130-11_ovis | lamb brain; 2013 | Sanger | KY828207 |
|  | PL_12_25_culic | *C. obsoletus/scoticus* complex parous; 2012 | Sanger | KY828208 |
| M (fragment corresponding to 1-188 aa of Gn protein) | PL_12_27-2_culic | *C. obsoletus/scoticus* complex parous; 2012 | Sanger | MF993056 |
|  | PL_12_28-2_culic | *C. obsoletus/scoticus* complex parous; 2012 | Sanger | MF993057 |
|  | PL_12_31-2_culic | *C. obsoletus/scoticus* complex parous; 2012 | Sanger | MF993058 |
|  | PL_12_36E-2_culic | *C. obsoletus/scoticus* complex parous; 2012 | Sanger | MF993059 |
|  | PL_12_38-2_culic | *C. obsoletus/scoticus* complex gravid; 2012 | Sanger | MF993060 |
|  | PL_14_962-2_culic | *C. obsoletus/scoticus* complex blood fed; 2014 | Sanger | MF993061 |
|  | PL_12_36A-2_culic | *C. punctatus* gravid; 2012 | Sanger | MF993062 |
| M (fragment corresponding to 410- 687 aa of HVR) | PL_12_27-4_culic | *C. obsoletus/scoticus* complex parous; 2012 | Sanger | MF993071 |
|  | PL_12_38-4_culic | *C. obsoletus/scoticus* complex gravid; 2012 | Sanger | MF993072 |
|  | PL_12_36A-4_culic | *C. punctatus* gravid; 2012 | Sanger | MF993073 |
|  | PL_12_32-4_culic | *C. obsoletus/scoticus* complex parous; 2012 | Sanger | MF993074 |
|  | PL_12_36E-4_culic | *C. obsoletus/scoticus* complex parous; 2012 | Sanger | MF993075 |
|  | PL_12_1016-32-4_bov | cattle serum; 2012 | Sanger | MF993076 |
|  | PL_12_1016-41-4_bov | cattle serum; 2012 | Sanger | MF993077 |
|  | PL_13_356-2-4_ovis | lamb brain; 2013 | Sanger | MF993078 |
| M (fragment corresponding to 1141-1394 aa of Gc) | PL_12_27-6_culic | *C. obsoletus/scoticus* complex parous; 2012 | Sanger | MF993063 |
|  | PL_12_38-6_culic | *C. obsoletus/scoticus* complex gravid; 2012 | Sanger | MF993064 |
|  | PL_12_36A-6_culic | *C. punctatus* gravid; 2012 | Sanger | MF993065 |
|  | PL_12_36E-6_culic | *C. obsoletus/scoticus* complex parous; 2012 | Sanger | MF993066 |
|  | PL_12_32-6_culic | *C. obsoletus/scoticus* complex parous; 2012 | Sanger | MF993067 |
| L (complete) | PL_12_1018-08_bov | cattle serum; 2012 | NGS | KY828193 |
|  | PL_13_130-6_ovis | lamb brain II passage BHK-21; 2013 | NGS | KY828194 |
|  | PL_12_1016-32_bov | cattle serum; 2012 | Sanger | KY828195 |
| L (fragment corresponding to 1137-1390 aa) | PL_12_27-11_culic | *C. obsoletus/scoticus* complex parous; 2012 | Sanger | MF993068 |
|  | PL_12_36E-11_culic | *C. obsoletus/scoticus* complex parous; 2012 | Sanger | MF993069 |
|  | PL_12_36A-11_culic | *C. punctatus* gravid; 2012 | Sanger | MF993070 |
