# Supplementary material for: Comparison of Schmallenberg virus sequences isolated from mammal host and arthropod vector
Source: Virus Genes. 2018 Oct 19;54(6):792–803. doi: 10.1007/s11262-018-1607-7 (PMC6244546; doi:10.1007/s11262-018-1607-7)
Supplement: Supplementary file 2 — Supplementary material 2—Table 2 List of reference sequences used in the study (DOC 88 KB) [file 11262_2018_1607_MOESM2_ESM.doc]

| Strain name | Genbank accesion No. | Reference |  | Strain name | Genbank accesion No. | Reference |
| --- | --- | --- | --- | --- | --- | --- |
| segment S | | | | | | |
| BH80/11-4 | HE649914.1 | Hoffmann et al., 2012 |  | W12-6 | KT795160.1 | Coupeau et al., 2016 |
| Na1-CNS | KC139376.1 | Coupeau et al., 2013 |  | W12-7 | KT795158.1 |  |
| Na2-CNS | KC139379.1 |  |  | S11-3 | KT795157.1 |  |
| BH02/12-1 | KC108842.1 | Fisher et al., 2013 |  | W12-16-2/3 | KT795156.1 |  |
| BH03/12-3 | KC108844.1 |  |  | W12-11-1/2 | KT795155.1 |  |
| BH28/12-5 | KC108846.1 |  |  | W12-10-3/3 | KT795153.1 |  |
| BH37/12-2 | KC108848.1 |  |  | W12-14 | KT795152.1 |  |
| BH77/12-1 | KC108852.1 |  |  | S12-4 | KT795150.1 |  |
| BH127/12-16 | KC108854.1 |  |  | W12-12-2/2 | KT795149.1 |  |
| BH148/12-9 | KC108856.1 |  |  | W12-1 | KT795148.1 |  |
| BH197/12-3 | KC108860.1 |  |  | W13-3 | KT795146.1 |  |
| BH198/12-5 | KC108862.1 |  |  | S12-1 | KT795145.1 |  |
| BH199/12-5 | KC108864.1 |  |  | W13-1 | KT795144.1 |  |
| BH200/12-2 | KC108866.1 |  |  | W12-2 | KT795143.1 |  |
| BH231/12-1 | KC108868.1 |  |  | W13-4-2/2 | KT795141.1 |  |
| BH233/12-1 | KC108870.1 |  |  | S11-2 | KT795140.1 |  |
| BH237/12-4 | KC108872.1 |  |  | W13-4-1/2 | KT795139.1 |  |
| BH250/12-2 | KC108876.1 |  |  | W12-19 | KT795133.1 |  |
| BH336/12-1 | KC108878.1 |  |  | S12-7b-2/2 | KT795132.1 |  |
| BH619/12-1 | KC108882.1 |  |  | S11-1 | KT795129.1 |  |
| BH652/12-1 | KC108886.1 |  |  | S12-7b-1/2 | KT795128.1 |  |
| HL 1 | KC355456.1 | Hulst et al., 2013 |  | W13-5-1/2 | KT795123.1 |  |
| F6 | KC355459.1 |  |  | W12-15 | KT795118.1 |  |
| SBV/2013/TR/Krkl.1 | KP279304.1 | Tonbak et al., 2013- unpublieshed |  | W12-9-1/2 | KT795114.1 |  |
| BH652/12 | KP731866.1 | Wernike et al., 2015 |  | Ib An 5550 | HE795107.1 | Goller et al., 2012 |
| D495/12-1 | KP731867.1 |  |  | - | HE795104.1 |  |
| segment M including HVR | | | | | | |
| BH80/11-4 | HE649913.1 | Hoffmann et al., 2012 |  | BH250/12-2 | KC108877.1 | Fisher et al., 2013 |
| Na1-CNS | KC139368.1 | Coupeau et al., 2013 |  | BH336/12-1 | KC108879.1 |  |
| Na2-CNS | KC139372.1 |  |  | BH336/12-3 | KC108881.1 |  |
| BH02/12-1 | KC108843.1 | Fisher et al., 2013 |  | BH619/12-1 | KC108883.1 |  |
| BH03/12-3 | KC108845.1 |  |  | BH635/12-2 | KC108885.1 |  |
| BH28/12-5 | KC108847.1 |  |  | BH652/12-1 | KC108887.1 |  |
| BH37/12-2 | KC108849.1 |  |  | HL 1 | KC355455.1 | Hulst et al., 2013 |
| BH59/12-8 | KC108851.1 |  |  | F6 | KC355458.1 |  |
| BH77/12-1 | KC108853.1 |  |  | 200.2 | KM047429.1 | Hoffmann et al., 2015 [43] |
| BH127/12-16 | KC108855.1 |  |  | 175.2 | KM047428.1 |  |
| BH148/12-9 | KC108857.1 |  |  | 102.2 | KM047427.1 |  |
| BH174/12-2 | KC108859.1 |  |  | 100.3 | KM047426.1 |  |
| BH197/12-3 | KC108861.1 |  |  | 96.1 | KM047425.1 |  |
| BH198/12-5 | KC108863.1 |  |  | 79.4 | KM047423.1 |  |
| BH199/12-5 | KC108865.1 |  |  | BH619/12 | KP731871.1 | Wernike et al., 2015 |
| BH200/12-2 | KC108867.1 |  |  | BH652/12 | KP731872.1 |  |
| BH231/12-1 | KC108869.1 |  |  | D495/12-1 | KP731873.1 |  |
| BH233/12-1 | KC108871.1 |  |  | BH119/14-1/2 | KP731874.1 |  |
| BH237/12-4 | KC108873.1 |  |  | BH119/14-3/4 | KP731875.1 |  |
| BH248/12-1 | KC108875.1 |  |  | BH132/14 | KP731876.1 |  |
| segment L | | | | | | |
| BH80/11-4 | HE649912.1 | Hoffmann et al., 2012 |  | 102.2 | KM047420.1 | Hoffmann et al., 2015 [43] |
| Na1-CNS | KC139362.1 | Coupeau et al., 2013 |  | 96.1 | KM047418.1 |  |
| Na2-CNS | KC139365.1 |  |  | 79.4 | KM047416.1 |  |
| HL 1 | KC355454.1 | Hulst et al., 2013 |  | BH119/14-1/2 | KP731877.1 | Wernike et al., 2015 |
| F6 | KC355457.1 |  |  | D495/12-1 | KP731879.1 |  |
| BH80/11-4 | HE649912.1 | Hoffmann et al., 2012 |  | BH132/14 | KP731882.1 |  |
| 200.2 | KM047422.1 |  |  | Ib An 5550 | HE795105.1 | Goller et al., 2012 |
| 175.2 | KM047421.1 |  |  | - | HE795102.1 |  |
